# What Dance taught a Doctor: A resident’s perspective on the role of humanities in psychiatry training

**DOI:** 10.15694/mep.2018.0000185.1

**Published:** 2018-09-03

**Authors:** Deva Priya Appan

**Affiliations:** 1 Institute of Mental Health

**Keywords:** Psychiatric education, Bharatanatyam, Humanities, Empathy

## Abstract

This article was migrated. The article was marked as recommended.

This article is a personal account of the author’s experience in a project that integrated psychiatry with dance. The author, who is a psychiatry trainee and an Indian classical dancer, describes how this dance project, started in an attempt to raise awareness and reduce stigma towards mental illness became a personally fulfilling and enriching experience as a psychiatry trainee.

## Introduction

As a junior doctor interacting with patients and their families, I started to appreciate the extent of their difficulties and challenges in our society today. What stood out most for me was the stigma that patients with mental illness face. Hence I felt fortunate to pursue training in Psychiatry and be able to help this group of patients.

Historically, there has been a strong emphasis on the biomedical model of disease in most of medicine. Psychiatry too has laid its focus on biological constructs, especially since the discovery of the role of neurotransmitters, in particular, the dopaminergic pathways ( Carlsson and Lindqvist, 1963). While leaders in psychiatry argue for a practice that is grounded in ‘evidence base’ from genetics and neurosciences, a significant proportion of clinicians concern themselves with the humane care of the vulnerable populations they tend to.

It has been proposed that psychiatry trainees would benefit with the integration of psychiatry and medical humanities, as it may improve understanding of mental illnesses and support the implementation of more effective interventions ( Bhugra and Ventriglio, 2015). In a time where young doctors report record rates of burn-out and loss of purpose, the inclusion of humanities in the medical curricula may help promote empathic skills ( Schlozman, 2017). In this essay, I present my musings on a personal experience of how a classical dance production helped me rekindle my passion and regain some empathy.

As a psychiatry trainee working in various sub-specialties, I could see my passion wane and my empathy gradually erode. It is at this juncture that an opportunity came by. I became involved in a project that integrated psychiatry with dance, two of my passions. What started out as a project to create awareness and reduce stigma towards mental illness became a personally fulfilling and enriching experience as a psychiatry trainee.

## Project preparation

I have been learning and practicing Bharatanatyam, an Indian classical dance form, for the last 26 years. Bharatanatyam originated from the Hindu temples of Tamil Nadu, India, and its style is noted for its intricate footwork and sophisticated vocabulary of sign language, based on gestures of the hands and the use of the eyes and face muscles ( Kalpana, 2015).

I was intrigued to discover the intersection of these ‘two worlds’ over the last few years. It first started with a dance performance, where I played the part of a female character (Sita) who had been abducted by an evil king. As I studied her character closely, I realized she had a low mood with poor appetite and became hopeless about her situation. The climax of the dance scene was an attempt at suicide. Having learned about depression and interacted with depressed patients eased me into the role with conviction. This is when an idea to portray mental health issues through this dance form took shape in my mind. Soon after, a senior dancer approached me to play the role of a young woman with schizophrenia. The dance recital was to portray her journey as a lead character and her mother’s struggle to cope with it ( Salleh, 2016). The aim of the production was to raise awareness of mental health issues using dance as a medium. This resonated deeply with me and I agreed to be a part of the production.

My research into the area, especially with regards to dance productions with a mental health focus, yielded unsatisfactory results. Most of these were within the context of contemporary dance forms. The link between dance and mental illness was more of a psychotherapeutic use of movement and dance, like in Dance Movement Psychotherapy (DMP).

At the very beginning when the production was being conceptualized, I had worries and anxiety about how the show would unfold and wondered if I would do justice to the role. These feelings reduced over time as the production took form, with more clarity. I realized I had to learn more about the psychotic experience from patients themselves. Perhaps, it was the beginning of empathic curiosity, an important tool to develop empathy ( McEvoy P, Baker D, Plant R, Hylton K, 2013). I saw myself spending more time with my regular patients. At that moment, it dawned on me that while fulfilling the duty to treat patients with psychotic symptoms, I had overlooked *listening* to their stories, an essential part of their recovery. After speaking to various patients and peer support specialists, I realized that although each person’s experience may have differed, what was similar were the emotions they experienced. They had felt bouts of anxiety, anger, and fear during those psychotic experiences.

## What Dance taught me

As I could not convey the psychotic symptoms with speech, I had to rely on my expressions and body language. I felt that I had to embody the character completely. An important scene that portrayed stigma had the other dancers ridicule my character. This was achieved by the dancers encircling me while pointing at me and bringing their palms to block my face, a confrontational experience indeed. The musical accompaniment with repetitive chanting (‘Aval paithiyam’ meaning ‘She’s mad’ in tamil) made it more unsettling. I wonder if this could be what being stigmatized feels like. I felt that I learned about psychosis and stigma from a different viewpoint, almost a quasi first- person perspective. As I danced and at the height of psychosis in my character, I became almost oblivious to others around me, akin to a depersonalization experience.

This project helped me empathize better with my patients and their caregivers. I was extremely pleased with the comments from some caregivers who conveyed how touched they had felt at being understood. This production also helped me to assess my own emotions and be more self-aware. The dance in itself acted as a stress relief for me. It helped me to work in a team and take on ideas from others while working towards a common goal.

## Discussion

One of the arguments against humanities in medical education is that it is impractical to add formal exploration of the humanities to an already packed medical curriculum ( Schlozman, 2017). On a personal note, I did spend a significant amount of my own time at rehearsals and in the planning of the production. Some have taken the argument further noting that the value added by studying the humanities in medical education is relatively small compared to the cost to students in terms of time, energy and an already packed schedule. I beg to differ. It was an enriching and an educational experience for me, one that could not have been obtained in an institution or hospital.

Another argument against humanities in medical education is that it would be a challenge to standardize it in an education pursued by students from various backgrounds with differing abilities and interests. This perspective from an outcome-based medical education is up for debate. The purpose of medical education should be to create caring, compassionate doctors, who are able to hone their own individual strengths and abilities.

I recommend that where feasible, every trainee should be given a chance to choose humanities of their interest, so as to be able to incorporate it into their learning.

## Suggestions

Medical educators could strive to ensure the following principles are adhered to when designing curriculum to include Humanities in medical/psychiatric education:

1. Learner-centric: To choose the humanities of their interest, to allow intrinsic motivation and to teach resilience/adaptability; Methods suggested could include visual arts, literature, film and music for example.
2. Measurable outcomes: Measuring the intangible outcomes such as empathy and holistic understanding of illnesses could be a challenge. Perhaps an indirect measure through methods such as narrative reviews or reflective journals could be used and discussed with supervisors spanning the period of their training.
3. Protected time within the realms of training schedule to pursue these interests; this also send a message that educators are serious about this process

## Take Home Messages

Humanities should be included in medical education, especially in psychiatric education, to help promote the learner’s empathic skills, to create a more holistic understanding of mental illnesses and to help relieve stress thereby reduce burn out in the future.

## Notes On Contributors

**Dr. Deva Priya Appan** is a 4th-year Psychiatry Resident and works in Institute of Mental Health, Singapore. She is also an aspiring medical educator, who enjoys teaching medical students and what she learns in the process. She continues to pursue dance (especially Bharatanatyam) in her leisure time, a passion she has followed since her childhood.
