# Supplementary material for: What Dance taught a Doctor: A resident’s perspective on the role of humanities in psychiatry training
Source: MedEdPublish (2016). 2018 Sep 3;7:185. [Version 1] doi: 10.15694/mep.2018.0000185.1 (PMC10701813; doi:10.15694/mep.2018.0000185.1)
Supplement: Peer review history

# Reviewer report: Reviewer response for version 1

**DOI:** 10.21956/mep.19648.r29295

**Published:** 2019-07-21

**Authors:** Gary D. Rogers

**Affiliations:** 1 Griffith University

This review has been migrated. The reviewer awarded 4 stars out of 5 Thanks, Deva, for this beautifully written account of the way you made meaning, as a psychiatrist-in-training, from your experience of portraying characters with so-called ‘mental disorders’ within a particular cultural medium. Your insights about the value of the arts in educating doctors about the human condition are well taken. The paradox here, though, is that the doctors who perhaps most need to gain these perspectives are the ones who are least likely to do so spontaneously or recognise this need. Thus, those who stand to gain the most (both for themselves and their patients) may need more guidance or even compulsion and would be less likely to recognise what they need if invited to ‘choose humanities of their [own] interest’. All the very best for your future development as a *human* psychiatrist.

Reviewer Expertise:

NA

No decision status is available

# Reviewer report: Reviewer response for version 1

**DOI:** 10.21956/mep.19648.r29293

**Published:** 2018-09-07

**Authors:** Jonathan McFarland

**Affiliations:** 1 Sechenov University, Moscow

This review has been migrated. The reviewer awarded 4 stars out of 5 This is a very interesting article that I would definitely recommend to those interested in (re)introducing the humanities into medical education. The paper is passionately felt by the author, a 4th year Psychiatry Resident and is about how her personal experience in classical Indian dance restored her passion for her medical training and work. The author cleverly and succinctly explains what the aims of the dance project were, what it entailed and how she could link it with her work; as she says it involved her two passions, dance and psychiatry. What I really like about this paper, apart from the fascinating subject matter and the already mentioned enthusiasm, are the arguments for why the humanities should be included in the medical curriculum, along with some suggestions as to how. I completely agree with the author when she says, “ I recommend that where feasible, every trainee should be given the chance to choose humanities of their interest, so as to incorporate it into their learning”, and would emphasise two things – “of their interest” and “incorporate into their learning”. These two factors are critical; the humanities should be incorporated or integrated into medical professionals’ learning and all learners should choose something that enthrals them so as not to be an added burden. Once again, I wish to congratulate the author, and hope that many read and learn from her experiences.

Reviewer Expertise:

NA

No decision status is available

# Reviewer report: Reviewer response for version 1

**DOI:** 10.21956/mep.19648.r29297

**Published:** 2018-09-05

**Authors:** Gert Van Zyl

**Affiliations:** 1 University of the Free State

This review has been migrated. The reviewer awarded 4 stars out of 5 Interesting contribution to the topical debate of humanities and to practical implement the concept. Appreciated that it is linked with psychiatry as work experience. Application of the approach may also be of value in other areas of treatment.

Reviewer Expertise:

NA

No decision status is available

# Reviewer report: Reviewer response for version 1

**DOI:** 10.21956/mep.19648.r29296

**Published:** 2018-09-04

**Authors:** P Ravi Shankar

**Affiliations:** 1 American International Medical University

This review has been migrated. The reviewer awarded 5 stars out of 5 I thoroughly enjoyed reading this personal opinion. The author is not only a gifted dancer but also a good storyteller. Her story flows smoothly and keeps the reader engaged. The author describes succinctly how dance helped her gain a more personal understanding of mental illness and helped her identity better with patients and caregivers. She passionately puts forward the case for investing time and effort in the humanities. I look forward to more articles from the author!

Reviewer Expertise:

NA

No decision status is available

# Reviewer report: Reviewer response for version 1

**DOI:** 10.21956/mep.19648.r29294

**Published:** 2018-09-03

**Authors:** Trevor Gibbs

**Affiliations:** 1 AMEE

This review has been migrated. The reviewer awarded 5 stars out of 5 I very much appreciated reading this very personal view which eventually led to concrete suggestions. The author wrote the paper with clear deep conviction regarding the way that dance can become more that just therapy, by giving insight to the patients inner feelings through the process of "acting-out" . I have read and been involved personally in playing the patient, but dance seems to give a better insight and seems to mirror some of those inner feelings of the patient.I don't feel that I can add more to this short review other than suggest such a paper is not only recommended to those involving the Humanities but to psychiatry trainees as well

Reviewer Expertise:

NA

No decision status is available
